# The impact of transport, housing, and urban development interventions on older adults’ mobility: A systematic review of experimental and quasi-experimental studies

**DOI:** 10.1016/j.jth.2024.101859

**Published:** 2024-09

**Authors:** Thiago Hérick de Sá, Daniele Sudsataya, Andra Fry, Nazak Salehi, Aishwarya Katiki, Megan Mcleod, Greg Rathmell, Jon Cylus, Louise Lafortune, Tine Buffel, Patty Doran, Alana Officer, Huseyin Naci

**Affiliations:** a Demographic Change and Healthy Ageing Unit, Department for Social Determinants of Health, World Health Organization, Switzerland; b Department of Health Policy, London School of Economics and Political Science, UK; c LSE Library, London School of Economics and Political Science, UK; d European Observatory on Health Systems and Policies, UK; e Cambridge Public Health, University of Cambridge, UK; f Manchester Urban Ageing Research Group, University of Manchester, UK

## Abstract

**Background:**

Age-friendly cities and communities aim to enhance and preserve the functional abilities of older adults. This systematic review assesses the impact of interventions in transportation, housing, and urban development on the mobility of older adults.

**Methods:**

We systematically searched MEDLINE, Embase, CINAHL, Scopus, PsycINFO, and SocINDEX up to July 2022 to identify studies that evaluated the impact of transportation, housing, and urban development interventions on older adults' mobility. Only randomised controlled trials and quasi-experimental studies with control groups were included to establish a causal relationship between interventions and mobility outcomes.

**Findings:**

We included a total of 15 studies, of which six were randomised controlled trials. Included studies were conducted in high-income settings and employed diverse metrics to assess mobility outcomes. Among housing interventions, three studies examined the impact of assistive technology within home environments for frail older adults. Two of these interventions maintained functional status without improvement, while the third showed a significant decline in outcomes, with the control group faring even worse. Public transport interventions, focused on enhancing mobility through educational initiatives and policy revisions, consistently produced positive outcomes. Interventions related to driving training for older adults, including in-class and on-road assessments, demonstrated beneficial effects. Results from studies evaluating urban design interventions were more varied, with some enhancing mobility by making public spaces more accessible for older adults and others yielding mixed results following infrastructure changes.

**Interpretation:**

Interventions in the built environments of older adults, specifically targeting transportation, housing and urban development, have the potential to enhance mobility and related outcomes according to rigorously designed quantitative evaluations. Due to heterogeneity in how mobility is conceptualised in the literature, greater harmonisation in measurement of mobility would help us understand how the social and built environment contribute to maintaining and improving mobility in older adults.

**Funding:**

World Health Organization.

## Highlights

• Prioritising universal accessibility for transport and urban spaces may improve older adults’ daily activity and mobility.
• Future research would benefit from agreed upon definitions of mobility for standardised measurements and data homogeneity.
• At-home tech-based interventions assessed in our review only maintained current levels of older adult functional ability.
• Driving training interventions in our review increased driving knowledge and reduced risky road behavior in older adults.
• Interventions that incentivised physical activity through age-friendly public spaces improved the movement of older adults.

## 1 Introduction

Population ageing is one of the most significant trends of the twenty-first century, transforming the way people live, work, and experience their environments. Over the period of the Decade of Healthy Ageing (2021–2030) – a global concerted effort to “add life to years” – the number of people 60 years and older globally is projected to increase by 34% to 1.4 billion, and by 2050 80% of the population aged 60 years and older will be living in low- and middle-income countries (World Health Organization, 2020).

Healthy ageing is defined by the World Health Organization (WHO) as “the process of developing and maintaining the functional ability that enables wellbeing in older age” (World Health Organization, 2015). Functional ability comprises the health-related attributes that enable people to be and to do what they have reason to value. It is made up of the intrinsic capacity of the individual, relevant environmental characteristics, and the interactions between the individual and these characteristics. Intrinsic capacity is the composite of all the physical and mental capacities of an individual. To assess the impact of its actions at the national, regional, and global levels, the WHO considered intrinsic capacity and functional ability as outcome indicators of healthy ageing. Domains of functional ability include the ability to 1) meet their basic needs, 2) learn, grow and make decisions, 3) be mobile, 4) build and maintain relationships; and 5) contribute.

Designing age-friendly cities and communities can go a long way in creating environments that develop and maintain the functional ability that enables wellbeing in older age; it enables all older adults, even when experiencing intrinsic capacity loss, to continue to do the things they value (Rémillard-Boilard et al., 2020). This understanding is also reflected in the Decade of Healthy Ageing, which has as one (out of four) action areas the development of communities in ways that foster the abilities of older adults (Greenfield and Buffel, 2022). Environments comprise the contexts of an individual's life which, for older adults, include home, social relationships, neighborhoods, communities (rural, urban, unregistered settlements etc.) and broader policies (economic, social etc.). Specific environmental factors other than the natural environment include policies, systems and services related to transport, housing, social protection, streets and parks, social facilities, health and long-term care, politics, as well as products and technologies, people and relationships (friends, family, care givers), cultural and social attitudes and values (van Hoof and Marston, 2021). Investigating the role the environment plays in the lives of older adults can highlight the direct relationship between health and transport, as it is understood that active travel in later life not only improves physical health, but can help foster a sense of community belonging that counters the sedentary and isolated lifestyles of many older adults (Musselwhite et al., 2015).

Action to improve the lives of older adults, their families, and the communities in which they live will therefore have to include interventions in the social and built environments, including through Age-Friendly Cities and Communities programmes - an internationally recognised approach, created by the WHO in 2006 and widely adopted around the globe. The WHO Global Network of Age-Friendly Cities and Communities (formed in 2010) currently has over 1500 members in more than 51 countries and includes as one of its core missions the support for cities and communities to find appropriate innovative and evidence-based solutions to improve healthy ageing (World Health Organization, 2024). Since the establishment of the WHO Global Network, research in this area has contributed significant knowledge about the different strategies appropriate for building age-friendly cities and communities. Studies have, for instance, focused on the steps associated with the age-friendly process, including the planning (Greenfield, 2018), implementation (McDonald et al., 2018), and evaluation (Buckner et al., 2019) of programmes. Researchers have also examined the development of age-friendly initiatives in diverse settings, encompassing both rural (Menec et al., 2015) and urban environments (Buffel and Phillipson), as well as in different countries (Moulaert and Garon, 2016, Rémillard-Boilard et al., 2020), with studies from low- and middle-income settings highlighting how such initiatives need to be sensitive to the everchanging urban contexts in which they are implemented (Woolrych et al., 2022), particularly due to older adults often encountering poor transport accessibility in these regions (Brugulat-Panés et al., 2023). Yet, evidence on the impacts of interventions in the social and built environments is limited for all domains of functional ability (Cochrane Global Ageing, 2024), including the ability to be mobile. A Cochrane systematic review and gap map, which assessed health, social, and technological interventions for the improvement of older adult functional ability, did not identify a single intervention related to transportation or homemaking; most of the evidence on interventions to improve mobility identified in this map is limited to frail older people and related to healthcare (Cochrane Global Ageing, 2024).

Mobility refers to “movement in all its forms, whether powered by the body (with or without an assistive device) or a vehicle” (World Health Organization, 2015) and is understood not only in relation to one's physical performance but also to one's ability to move in time and space as part of the everyday life activities. Therefore, interventions to improve mobility will go beyond those specifically focused on improving physical performance (e.g., rehabilitation) to include those related to key determinants of older people's wellbeing, such as transport, housing, and urban development. In this sense, it is still unclear what the best interventions are to support people's mobility in later life, particularly interventions in other domains of action of age-friendly cities and communities (beyond healthcare, and with older people living in the community) (World Health Organization, 2007).

Research on older adult mobility and its impact on health is growing. Previous systematic reviews have assessed the impact of the built environment on various aspects of older adults' quality of life. One review exploring the impact of the built environment on loneliness found that neighborhood walkability, transportation access, housing and urban planning all had the potential to combat loneliness in older adults (Lyu and Forsyth, 2022). Another review used older adult wellbeing as an overarching framework to evaluate whether older adults had a good fit with the built environment they resided in, and if this had an effect on their mobility. This review highlighted how the current body of literature tends to focus on built environment and physical activity interventions for older adults, often disregarding the effect on quality of life and improvements at a holistic level (Li, 2020). Finally, one review found that existing research does not often consider or attempt to measure accessibility and accessibility-centric interventions' effects on older adults’ health (Li, 2024). To our knowledge, there are no reviews explicitly focused on experimental and quasi-experimental studies evaluating the effectiveness of transportation, housing, and urban design interventions in improving the functional ability of older adults. Moreover, there is a paucity of literature that specifically attempts to quantify the effects of such interventions on their mobility. To fill this evidence gap, we performed a systematic review to assess the impact of transport, housing and urban development interventions on the mobility of older adults.

## 2 Methods

Our review question was: what is the impact of transport, housing, and urban development interventions on the mobility of community-dwelling older adults? We sought to identify studies that evaluated the causal impact of transport, housing, and urban development interventions on the mobility of older adults. As our objective was to systematically search for, appraise, and synthesise the relevant body of literature, we adopted a systematic review approach (Grant and Booth, 2009). The protocol for the review was registered prior to title and abstract screening (CRD42022343905). The review is reported according to the PRISMA guidelines (Page et al., 2021).

### 2.1 Eligibility criteria

*Participants/population:* The review focused on older adults living in the community. For this review, we accepted operational definitions for "older adults” from 50 years old onwards, considering that intervention studies commonly recruit participants at earlier ages to account for the lag between the intervention and its impacts. We considered participants from cities and communities of any size and location in urban, peri-urban and rural areas.

*Interventions(s)/exposure(s):* We focused on interventions related to environmental areas closely related to mobility. Namely, we included any transport, housing, and urban development intervention with primary data on the impact on the mobility of older adults. We sought to identify both interventions specifically targeted at improving the lives of older adults (e.g., public transport fare exemption for people aged 60 and over) and interventions with a broader target population that report the impact on older adults’ mobility (e.g., interventions for accessible transportation).

*Comparator(s)/control:* Studies that included any type of comparators/control were eligible for inclusion. Studies without a comparator were excluded.

*Main outcome(s):* The main outcome of interest was the older adults' mobility, one of the domains of functional ability. The conceptualisation of functional ability proposed by the WHO considers an individual and their interactions with their environments as inherently linked to what people can be and do. The intrinsic capacity of an individual depends on their physical and mental capacities. When an older adult's intrinsic capacity declines such that it reduces their functional ability, including the ability to be mobile, it is precisely the environment that can compensate for these difficulties and support an individual (World Health Organization, 2015). In the case of mobility, interventions in the built environment do not need to specifically target an individual's ability to be mobile. Rather, they can still increase mobility by simplifying other aspects of life (for example by rendering a neighborhood more safe), and as such indirectly promoting movement (World Health Organization, 2015). As previously mentioned, mobility “refers to movement in all its forms, whether powered by the body (with or without an assistive device) or a vehicle”*,* including sub-domains such as getting up from a chair or moving from a bed to a chair, walking for leisure, exercising, completing daily tasks, driving a car and/or using public transport (World Health Organization, 2015). We used this definition to identify eligible studies and did not restrict outcomes identified by the sub-domains mentioned above. We also considered studies that included older adults' perception of their mobility status. Adopting this broad definition of mobility ensured we did not exclude relevant studies solely due to a lack of a specific mobility measure.

*Additional outcome(s):* We sought to capture additional outcomes reported in included studies, such as, well-being, mental health, independence and outcomes falling under the broad umbrella of social inclusion, connectedness, belonging.

*Study design:* Study eligibility was limited to designs that could demonstrate a causal link between the intervention and mobility outcomes. In addition to experimental studies (i.e., randomised controlled trials), quasi-experimental design studies were eligible if the assignment of participants was based on allocation rules such as alternate assignment (quasi-randomised studies), inclusion of a threshold on a continuous variable (regression discontinuity designs), or exogenous variation in the treatment allocation (natural experiments) or other rules including self-selection by investigators or participants, provided that data were collected contemporaneously in a comparison group (non-equivalent comparison group design), or an interrupted series design with at least three data points both before and after a discrete intervention (six-period interrupted time series) (Reeves et al., 2017; Shadish et al., 2002).

### 2.2 Literature search and study identification

#### 2.2.1 Scoping searches

As recommended in the Cochrane Handbook for Systematic Reviews of Interventions, an information specialist provided advice on and then developed the search strategies for this review. An information specialist is an expert in designing and executing comprehensive search strategies across multiple information sources, ensuring the search process is thorough and methodical (Higgins et al., 2023). Their aim is to find all the relevant studies and minimise bias, which is crucial for the integrity of a systematic review. To start with, we performed a scoping search to identify whether there was sufficient literature examining the influence of social and built environment healthy-ageing interventions on older persons’ mobility, and to inform the search strategy for the systematic review. We initially combined search terms related to sectors and interventions related to transport, housing, urban development, health and long-term care, information and communication, education and labour, and social protection and assistance, with terms related to old age and mobility.

Our preliminary scoping searches were structured around interventions with evidence of impact on the mobility domain of functional ability, guided by individual specifications of environmental factors (e.g., getting up from a chair; moving from a bed to a chair, completing daily tasks, exercising; driving a car or using public transport; and accessing shops, services, social and cultural activities and facilities in the community, walking for leisure, exercising). Searches were aimed at finding outcome-focused papers assessing relevant interventions; special focus was placed on identifying related systematic reviews to further highlight the need of performing this systematic review and to identify eligible papers. We performed preliminary searches on Scopus between May 26, 2022, and June 9, 2022.

For preliminary scoping searches, the search strategies were restricted to yield studies with at least title and abstract in English, as well as limiting results according to the following Scopus subject areas: Medicine, Social Sciences, Nursing, Psychology, Environmental Studies, Health Professions, Economics, Econometrics and Finance, Multidisciplinary, and Engineering.

#### 2.2.2 Systematic review search strategy

For the systematic review, we conducted searches in six bibliographic databases: MEDLINE Ovid, Embase Ovid, PsycINFO Ovid, CINAHL EBSCO, SocINDEX EBSCO and Scopus. A comprehensive search strategy was developed by an information specialist for the following concepts and logic: older adults AND mobility AND intervention evaluations AND (transport OR housing OR urban development). The search strategy included keyword searches in the title, abstract, and author keywords fields, using operators like truncation and proximity. Searches were conducted on June 29, 2022. The search strategy was adapted for each database. Where possible, search filters were used to exclude animal studies, as well as publication types like comments, editorials, or letters. No language or date limits were imposed on the search. We deduplicated records in EndNote 20 using the Falconer and Bramer methods (Falconer, 2018; Bramer et al., 2016). We also searched the UN Decade Knowledge Platform and the WHO Global Database of Age-Friendly Practices, and checked references of the included studies to identify additional relevant literature. The full search strategies for all databases are presented in Appendix 1.

### 2.3 Study selection

Eligible studies had to meet all the following criteria: evaluate interventions related to environmental areas closely related to mobility (Transport; Housing; Urban Development) and include older adults living in the community with a cut-off of 50 or over, or reported results separately for older persons aged 60 years or over. We excluded the following records: only in abstract format or conference proceedings; with title and abstract not published in English; and reviews. Reviews were kept to manually check reference lists.

Using Rayyan, a software application tailored for systematic reviews, two reviewers independently performed the title and abstract screening according to review eligibility criteria and performed full-text screening of all records included by any reviewer at the title and abstract screening stage. Disagreements were resolved by a third reviewer.

### 2.4 Data extraction

Two reviewers independently extracted data using a pre-designed and pilot-tested data extraction sheet. We extracted the following information: name of the ﬁrst author; publication date; country(ies) and Low-to-Middle-High-Income (LMHIC) status according to World Bank Data; study design; characteristics of the population (e.g., mean or median age, sample size, sex, rural and urban); intervention details (e.g., name, type, duration, costs, etc.); follow up of outcome measurement; findings; definition and operationalisation of mobility; involvement of older adults in the study, any other outcomes. The two reviewers discussed to resolve any disagreements and consulted with a third reviewer when needed.

### 2.5 Quality assessment of included studies

One reviewer evaluated the quality of each eligible study using the Cochrane risk of bias tools for randomised and non-randomised studies (Sterne et al., 2016, 2019). Uncertainties were resolved through discussion with another reviewer. No studies were excluded on the basis of quality assessment.

### 2.6 Synthesis of results

We summarised our major review findings descriptively using tables and plots, as well as narratively. We had originally planned to perform meta-analyses to pool the effects or provide median, minimum, and maximum values for the estimates. However, this was not possible because there was substantial conceptual and methodological heterogeneity in study designs, interventions, and outcome measures.

## 3 Results

### 3.1 Eligible studies

**Fig. 1:**
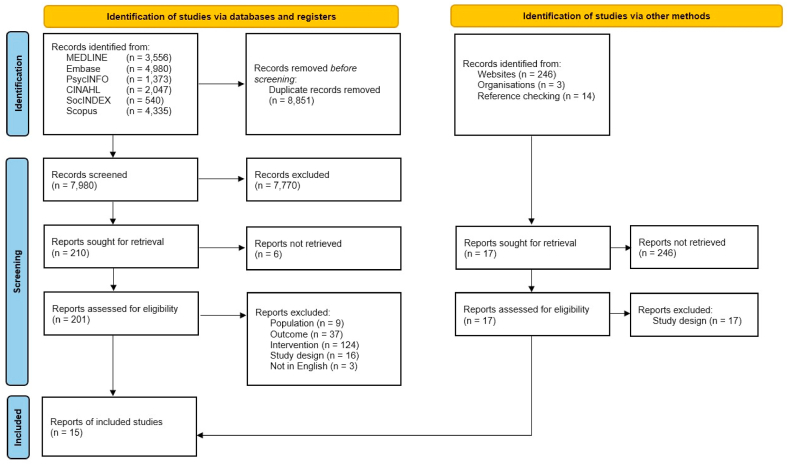
PRISMA flow chart for review selection process.

The database searches yielded 16831 studies. Following deduplication, 8851 records were removed, with 7980 studies screened in the title-abstract phase. Of these, 201 records were screened in the full text phase. In total, 15 studies were eligible for inclusion. Additionally, 17 studies retrieved via other methods were assessed for eligibility, but none were included. Fig. 1 shows the full selection process, including exclusion reasons for full text records.

### 3.2 Study characteristics

Six of the included studies had experimental designs (randomised controlled trials), and nine had quasi-experimental designs (Table 1). Included studies were conducted in various high-income countries such as the US, UK, France, Australia, among others. Three studies evaluated housing interventions, six evaluated transportation interventions (comprising three studies evaluating public transport interventions and three studies evaluating driving training interventions), and six studies focused on urban design interventions (see Table 2).

**Table 1:** Characteristics of included studies.

Study Name	Year	Location	Population	Time	Setting	Mobility Outcome
Tomita et al., 2007 (Housing)	2007	US	Frail older adults living alone	2 years	Home	Physical status
(Dupuy and Sauzéon, 2020) (Housing)	2020	France	Frail individuals >70	9 months	Home	Frail older individuals' functional status
Mann et al., 1999 (Housing)	1999	US	Frail individuals	18 months	Home	Functional status
Whitley et al., 2020 (Public Transport)	2020	UK	3 age groups (50–59, 60–64, 65–74)	14 years	England	Bus use, useability, social participation, satisfaction
Di Stefano et al., 2009 (Public Transport)	2009	Australia	60+	4 weeks	Several communities	Self-reported planned changes to mobility behavior, knowledge of driver assessment availability, knowledge of education program topics
Broome et al., 2013 (Public Transport)	2013	Australia	Urban older populations	3 years	Urban areas	User satisfaction and repeated use
(Bédard et al., 2008) (Driving)	2008	Canada	65+ drivers	2 sessions of 3–4 h	3 communities	The safe driving knowledge questionnaire, on-road driving exam
Marottoli et al., 2007 (Driving)	2007	US	Community living 70+ via Veterans Administration in Connecticut	8 weeks	Connecticut	Performance on knowledge and on-road tests
Edwards et al., 2009 (Driving)	2009	US	60+	3 years	Alabama and Kentucky	Driving difficulty
Thompson et al., 2014 (Urban Design)	2014	UK	65+	2 years	Community	Overall activity levels, health, quality of life
Hallgrimsdottir et al., 2015 (Urban Design)	2015	Sweden	65+	3 years	Community	Frequency of walking, frequency of activity
Frank et al., 2021 (Urban Design)	2021	Canada	4-65 and 65+	3 years	Community (near green way)	Use of urban greenway for healthy and active lifestyle (e.g., bicycling activity)
Prins et al., 2019 (Urban Design)	2019	Netherlands	67.5 avg	3 months; 9 months	Poor residential neighborhood	Total time spent walking per week, time spent utilitarian walking per week, time spent recreational walking per week
Marquet et al., 2017 (Urban Design)	2017	Spain	65-75 and 75-85	10 years	Community (Barcelona)	1) Immobility 2) time invested in active modes of transport 3) activity 4) time spent driving 5) time spent in transportation 6) share of people who solely through transportation achieved the WHO and CDC Physical Activity recommendations
Carp, 1979 (Urban Design)	1979	US	72.2 avg; qualified for public housing	6 months	One building within a community	% of pastimes that were active, self-reported assessment of activity level

**Table 2:** Results of included studies.

Study Name	Intervention Aims	Results Summary	Direction and Size of Effect
Tomita et al., 2007 (Housing)	Use of X10-based smart home technology for older adults vs. controls	Participants benefited from the smart home technology, and 91% recommended its use by others. The treatment group maintained physical and cognitive status, whereas the control group declined significantly in both.	Toward intervention. Scores flat with intervention and worsening with control
(Dupuy and Sauzéon, 2020)(Housing)	Use ambient assistive technology tools to increase older adult everyday functioning	Frail older individuals autonomy reported by caregivers is improved over time for the intervention group, with a greater extent after 9 months; "Comparisons showed that according to caregivers, the equipped group's autonomy remains constant from t0 to t9; whereas the score decreased significantly between t0 and t6 for the control group (p < 0.001)."	Toward intervention. Scores flat with intervention and worsening with control
Mann et al., 1999 (Housing)	Participants received home environmental interventions and assistive technology	After the 18-month intervention period, the treatment groups showed a I significant decline for the Functional Independence Measure total score and Functional Independence Measure motor score, but there was significantly more decline for the control group. Functional Status Instrument pain scores increased significantly more for the control group.	Significantly toward intervention. Scores flat with intervention and worsening with control
Whitley et al., 2020 (Public Transport)	Rise in eligibility age for bus passes	“Less than 20 per cent of 50–59 year olds reported using buses weekly and this was broadly consistent over time. Weekly bus travel rates were higher in 60–64 and 65–74 year olds, increasing in both groups when travel became free (yearly change: 2.6% (95% CI = 0.0, 4.9%) and 1.2% (95% CI = −0.1, 3.5%) in those aged 60–64 and 65–74 versus 50–59, respectively; p = 0.09). Weekly bus travel decreased in both groups from 2010 onwards (yearly change = −2.9% (95% CI = −4.I, −1.7%) in both those aged 60–64 and 65–74 versus 50–59; p < 0.001).”	Toward the control (note intervention); moderate size
Di Stefano et al., 2009 (Public Transport)	Education programs on mobility choices	“There was a significant increase in knowledge from before to after the program for 4 items considering the subgroups of all the participants and for 3 items for the participants completing all 3 stages of evaluation."	Toward interventions; some impacts to choices
Broome et al., 2013 (Public Transport)	Age friendly guidelines: lower floor buses, driver training, ped infrastructure, more buses, bus buddy	“After the intervention, the samples in Hervey Bay and Brisbane differed significantly in the proportion of participants who currently drove (X2 = 5.372,p = 0.020) with a smaller proportion of current drivers in the city of Brisbane. After intervention, the proportion of participants who experienced a disability that made it difficult to catch a bus was significantly lower in Hervey Bay than Brisbane post-intervention (X2 = 8.354,p = 0.004) or Hervey Bay pre-intervention (X2 = 5.488,p = 0.019)."	Toward intervention; large effect
(Bédard et al., 2008) (Driving)	In class and on road training	“This analysis revealed a statistically significant improvement after the intervention (t = 5.72, p < 00.001). The change observed is equivalent to an increase from 61% of questions correctly answered at baseline to 81% at follow-up."	Toward intervention. Large effect
Marottoli et al., 2007 (Driving)	Classroom and on road driving training	The least squares mean change in road test score relative to baseline was 2.87 points higher in the intervention than in the control group (p = 001). The least squares mean change in knowledge test scores relative to baseline was 3.45 points higher in the intervention than in the control group (p = 00.001).	Toward intervention. Moderate effect
Edwards et al., 2009 (Driving)	Computer-based Speed of Processing Training (SPT) on processing speed	“No significant effects were found in intention-to-treat analysis. However, number of SPT sessions did affect driving mobility outcomes. In the full sample, higher SPT doses were associated with maintained driving frequency as compared with both control groups, but no effects were found for driving exposure or space. Subsample analyses (n = 315) revealed that persons at-risk for mobility declines (i.e., poor initial processing speed) who received additional booster SPT sessions reported greater maintenance of both driving frequency and exposure over time as compared with the no-contact and active control groups.”	Toward intervention. Some reduction of decline
Thompson et al., 2014 (Urban Design)	Clean, nuisance-free local park, attractive, barrier-free routes and other natural environments	“Participants in the intervention group perceived that they were more active post intervention than 2 years previously, significantly more so than those in the comparison group (p = 0.04) (differences based on *t*-test). There were some common trends across the two groups, whether the intervention or comparison group: a decline in QoL measure CASP-19 (p = 0.04) and an increase in the number of unhealthy days (p = 0.006)."	Toward intervention. Significant for perception of activity
Hallgrimsdottir et al., 2015 (Urban Design)	More benches, separation from bikes, lower curbs, traffic calming	“comparison between the respondents in Study Area (SA) and Reference Area (RA) showed that those living in SA had significantly higher activity level, both in terms of frequency of walking (P = 0.000) and frequency of activity (P = 0.000)." "Those that perceived their health as poor and living in SA were more likely to often participate in activities than their counterparts in RA (P = 0.029; OR = 2. 141); those not dependent on walking in SA were more likely to often participate in activities than their counterparts in RA (P = 0.000: OR = 2501)."	Toward intervention. Large effect
Frank et al., 2021 (Urban Design)	Construction of urban greenway	“7.5% of subjects in the experimental group used a bicycle to complete at least one trip at baseline, with a percentage decrease of 11.1% at follow-Up (t = 0.50. p = 0.618). In the control group, 7.7% of subjects used their bicycle at baseline, with a percentage increase of 18.2% at follow-up (t = 0.89, P = 0.372). Neither of the changes in bicycle use were significant."	Toward intervention. Not statistically significant
Prins et al., 2019 (Urban Design)	Designated walking path; walking group; combo	“total time spent walking per week increased between TO and TI for all conditions. The Incidence Rate Ratio (IRR) for the physical condition was 1.46 (95% CI: 1.06; 2.05) and for the social intervention 1.52 (95%CI: 1.07; 2.16). At T2 these differences remained significant for physical conditions only … results mirrored for utilitarian walking … no evidence was found for an effect on recreational walking"	Toward intervention. Not significant
Marquet et al., 2017 (Urban Design)	Walkability	Reported as Difference-in-difference-- > low-walkability areas: immobility = −0.5, # of Trips = 0.01, Total Minutes Walked = 9.6, Total Minutes Driving = −0.4, Total Minutes Travelling = 9.1, Meeting Physical Activity Guidelines = 8.8. High-walkability areas: immobility = 4.3, # of Trips = 0.19, Total Minutes Walked = 9.7, Total Minutes Driving = 1.3, Total Minutes Travelling = 8.9, Meeting Physical Activity Guidelines = 11.5	Toward intervention. Large effect
Carp, 1979 (Urban Design)	Opportunity for activities	In general, applicants who were more active and who expressed interest in additional specific activities showed greatest gains in activity after moving; while the new environment seemed to confirm inactivity on the part of those originally least active. The data document the existence of "latent demand” for activity.	Toward intervention. Some improvements

Two studies assessed a more general population, but were included in this review as they reported results for older persons (aged 60 and above) separately (Whitley et al., 2020; Frank et al., 2021); the remaining thirteen studies exclusively assessed older adults. The duration of included studies varied substantially: six interventions lasted a minimum of 3 years with the longest running for 14 years (Whitley et al., 2020), while nine interventions were completed within 2 years, with the shortest conducted over 2 sessions lasting 3–4 h each (Bédard et al., 2008).

The types of interventions, as well as their stated aims, varied across the included studies. All evaluations of housing interventions looked at how at-home assistive technology could impact the functional and physical status of frail older populations (Mann et al., 1999; Tomita et al., 2007; Dupuy and Sauzéon, 2020).

Out of the three studies evaluating public transport interventions, one assessed the impact of increasing the eligibility age for bus passes (Whitley et al., 2020), one looked at educational programmes and their effect on safe public transport choices (Di Stefano et al., 2009), and the last of these assessed various infrastructural changes to make buses adhere to age friendly guidelines (Broome et al., 2013). There were three studies evaluating driving interventions, two of which evaluated the impact of in-class and on-road driving training (Bédard et al., 2008; Marottoli et al., 2007), and the other focusing on computer-based training to improve the visual processing speed of older adults (Edwards et al., 2009).

Of the six studies evaluating urban design interventions, three studies assessed the impact of infrastructural changes to community spaces to enhance safety and mobility, for example through a dedicated cycling greenway, barrier-free routes in parks, and more benches (Frank et al., 2021; Thompson et al., 2014; Hallgrimsdottir et al., 2015), two studies evaluated interventions aimed to improve and promote walkability (Marquet et al., 2017; Prins et al., 2019), and one study evaluated an intervention that provided opportunities for social gathering and leisure pastime activities within the community of a nine-story residential building (Carp, 1979).

### 3.3 Risk of bias assessment

Only one out of the six included randomised controlled trials was judged to be at low risk of bias (Bédard et al., 2008). All other included experimental studies were instead at either high risk of bias or marked as having some validity concerns. Only two of the included quasi-experimental studies were judged to be at low risk of bias (Whitley et al., 2020; Di Stefano et al., 2009). The remaining studies had either moderate or serious validity concerns.

Appendix 2 shows the risk of bias assessment for included experimental studies and quasi-experimental studies. In the experimental studies, Tomita et al. (2007) and (Hallgrimsdottir et al., 2015) were both classified as having high-risk of bias due to limitations in their randomisation process, while also raising some concerns in the measurement of the outcome domain; the latter study also had some concerns surrounding the selection of the reported result (Tomita et al., 2007; Hallgrimsdottir et al., 2015). Mann et al. (1999) also showed some concerns within its randomisation process, but was flagged as having a high risk of bias due to its outcome measurement (Mann et al., 1999). As for the quasi-experimental studies, Carp (1979) was deemed to have serious validity concerns due to selection bias, with moderate concerns regarding its classification of interventions and outcome measures, while Prins et al. (2019) also had serious validity concerns due to its outcome measurement (Prins et al., 2019; Carp, 1979). Finally, Thompson et al. (2014) was judged as having serious validity concerns overall due to moderate concerns regarding its result selection, outcome measurement, and missing data (Thompson et al., 2014).

### 3.4 Synthesis of results

#### 3.4.1 Housing interventions

Two of the at-home assistive technology interventions succeeded in maintaining, but not improving, the physical and cognitive status, as well as the autonomy, of older adults; their respective control groups showed significant decline in these outcomes (Tomita et al., 2007; Dupuy and Sauzéon, 2020). Participants of the Tomita et al. study were satisfied with the assistance provided by the intervention, with 91% recommending the smart home technology (Tomita et al., 2007). In terms of mobility measurements, Tomita et al.’s study reported effects on mobility using the Craig Handicap Assessment and Reporting Technique (CHART), whereas Dupuy and Sauzéon (2020) captured any impact on mobility through the Instrumental Activities of Daily Living (IADL) scale, where higher scores constituted greater reported difficulties in daily activities. The 1999 study by Mann et al. instead showed a significant decline in both the Functional Independence Measure total score and Functional Independence Measure motor score following the intervention, but these declines were even more significant in the control group (Mann et al., 1999).

#### 3.4.2 Public transport interventions

The three included public transport interventions aimed to improve mobility through education and the revision of guidelines and policies. Di Stefano et al. (2009) evaluated the effect of a community mobility training programme (Transport Accident Commission Community Mobility program for Older People), on the mobility-related decision-making of older adults, and found that such mobility choice education programmes led to significant increases in knowledge and knowledge retention regarding eyesight checks and issues linked to driving with a disability; these were measured using a pre-program and post-program questionnaire, as well as telephone interviews (Di Stefano et al., 2009). Broome et al. (2013) assessed the impact of a series of age-friendly guidelines for buses, including lower floor buses, flexible routes, bus buddy programmes, as well as more accessible pedestrian infrastructure. Participants used a 5-point Likert scale to record their degrees of satisfaction, which enabled the authors to capture changes in satisfaction that could in turn influence mobility-related outcomes. They found that bus driver age-awareness training led to an increase in perceived friendliness and in helpfulness, leading to higher satisfaction (Broome et al., 2013). Broome et al. (2013) further reported that their age-friendly bus guidelines had a large effect on mobility, with the proportion of participants experiencing a disability that made bus travel complicated being significantly lower in the intervention area than in a comparator area (Broome et al., 2013).

The third included public transport intervention, Whitley et al. (2020), evaluated the impact on older adult mobility following the rise in eligibility age for senior bus passes for concessionary travel. This was done using the UK National Travel Surveys data and weights which allowed for the evaluation of travel pattern variation over time. The study found that free bus rides led to increases in bus usage in older adult groups, however, raising the eligibility age for bus passes decreased bus use in all age groups (−2.9%) (Whitley et al., 2020).

#### 3.4.3 Driving

All three studies under the driving category evaluated interventions that aimed to improve older adult mobility through some form of training course. The training programmes assessed in Bédard et al. (2008) and Marottoli et al. (2007) were both in-class and on-road, and measuring performance on knowledge and on-road test, The driving training assessed in Bedard et al.’s study, which measured outcomes through a safe driving knowledge questionnaire completed both before and after the in-class training, as well as an on-road evaluation that standardised performance scores based on the Province of Manitoba evaluation procedure, led to improvement post intervention (t = 5.72, p < 00.001), with a 20% increase in correctly answered test questions (Bédard et al., 2008). Marottoli et al. (2007) instead based their in-class assessment on the AAA driver improvement program and their on-road test on the Connecticut Department of Motor Vehicles, which evaluated driving abilities in different settings. They found that their training led to moderate increases in on road test (2.87 points higher compared to control) and knowledge test (3.45 points higher compared to control) performances (Marottoli et al., 2007).

Edwards et al. (2009) used a computer-based training software to measure visual processing speed and driving difficulty. Mobility outcomes were captured in the study's Mobility Driving Habits Questionnaire, which assessed driving behaviours over a seven day to two-year long time period. This intervention yielded no significant findings in the intention-to-treat analysis, but found that older adults at higher risk of mobility-declines who participated in a greater number of speed of processing trainings showed better maintenance of both driving frequency and driving exposure over time when compared to control groups (Edwards et al., 2009).

#### 3.4.4 Urban design

Thompson et al. (2014) and (Hallgrimsdottir et al., 2015) both evaluated interventions that aimed to make public spaces safer and more accessible for older adults through the creation of barrier-free routes, installation of more benches, separation of cycle lanes and other safety-risks, and lower curbs. The Thompson et al. study measured mobility outcomes through a multiscale questionnaire that included mobility-related dimensions such as a walking as a secondary outcome, and a physical activity assessment by accelerometry using an Actigraph GT1M; the intervention was found to have significant impact on the perceptions of older adults, with participants stating they were more active post intervention than the 2 prior years, significantly more than those in the comparison group (p = 0.04) (Thompson et al., 2014). The urban design changes evaluated in Hallgrimsdottir et al., observed through a questionnaire where respondents’ self-reported functional limitations and dependence on mobility devices were evaluated using the Housing Enabler instrument, were also found to have a large effect on their specified mobility outcomes, with participants living in the study area showing significantly higher frequency of walking (p < 0.001) and activity (p < 0.001) (Hallgrimsdottir et al., 2015).

The study by Frank et al. (2021), which assessed the impact of a dedicated urban greenway for cycling on overall mobility for people of all ages, including specific results for older adult groups, did not find any significant changes in observed bicycle usage. Mobility-related outcomes were captured in a two-day travel behaviour diary, which used bicycle use and cycling trip frequency as outcome measures (Frank et al., 2021).

The 2019 study by Prins et al. and the 2017 study by Marquet et al. looked at how infrastructural changes that promoted walkability, such as a designated walking path, could help improve and incentivise the mobility of older adults. Prins et al.’s intervention, which measured walking through the International Physical Activity Questionnaire, did not have an effect on its targeted mobility outcome of recreational walking (Prins et al., 2019); however, the intervention in Marquet et al., which used a walkability index that considered walkable land-uses versus non-walkable uses, had a large effect on mobility, with areas with high walkability having a positive effect on mobility and activity maintenance (Marquet et al., 2017).

Lastly, the Carp (1979) study included some form of social environment intervention that focused on community-based walking and promotion of physical activity opportunities in groups, which overall aimed to improve mobility and healthy behaviours by bringing together groups of older adults to support each other in their physical activity. Mobility outcomes were observed through latent-demand, or in other words the difference in activity rate before and after the addition of opportunities. This intervention led to some improvements in physical activity participation, but only in those who were already more active at baseline (Carp, 1979).

## 4 Discussion

### 4.1 Key findings

This systematic review synthesised the scientific literature on the effectiveness of housing, transportation, and urban design interventions on the mobility of community-dwelling older adults, providing quantitative evidence to support the development of future healthy-ageing policies.

Overall, for housing studies, the at-home assistive device and technology-centered interventions did not lead to any statistically significant improvements in their targeted mobility domains, but rather, they succeeded in maintaining current levels of functional ability among older adults, especially when compared to the significant declines observed in each study's control groups. These results can be at least partially explained by the sample characteristics, mainly composed of frail older adults. Therefore, preserving current functional levels can be interpreted as a positive outcome of the interventions, as they prevent the detrimental consequences of declining functional abilities in frail older adults.

Limited but promising findings from our review around transport include, for example, the study by Broome et al. (2013), which found that age-friendly bus modifications led to improved older adult satisfaction, but did not have significant effects on mobility (Broome et al., 2013). This intervention alone may not improve older adult mobility as effectively as fee-reduction strategies, such as the one evaluated in the study by Whitley et al. (2020) which measured in a more direct manner the links between older adult bus passes and variations in travel, therefore mobility, patterns (Whitley et al., 2020). A combination of both may lead to better results, as it is likely that increased satisfaction can prompt more consistent use of the service when paired with discounted prices. The interventions evaluating driving training courses, such as those in Marottoli et al. (2007) and (Bédard et al., 2008), were found to increase driving knowledge and reduce risky driving behavior in older adults, prolonging their ability to drive independently and maintaining their immediate mobility, measured through safe-driving knowledge and driving behaviours, when compared to control groups (Bédard et al., 2008; Marottoli et al., 2007). Considering the negative effects of ageing on driving ability (ex. speed of processing), training interventions may lead to promising improvements in mobility, or at the very least, prevent safety concerns and delay reductions in functional ability. At the same time, little can be said regarding the impacts of the interventions on knowledge and safer driving behaviour and ultimately on the mobility of older people over a prolonged period of time. There is also limited evidence from experimental or quasi-experimental studies on whether car-based travel patterns are overall worse for older people as compared to travel patterns reliant on public transport, walking and cycling, as indicated by health impact assessment studies (Mueller et al., 2015).

Urban design studies yielded encouraging results, as evaluated in Hallgrimsdottir et al. (2015) and (Marquet et al., 2017): interventions that incentivised physical activity through age-friendly infrastructure and removal of safety hazards in public spaces were found to be simple yet effective strategies to improve the day-to-day movement of older adults, as measured in walkability indexes or as solutions to self-reported functional limitations (Hallgrimsdottir et al., 2015; Marquet et al., 2017). Urban design studies were also the ones most commonly evaluating a package of interventions rather than a single intervention in isolation, which better reflects the approach taken by e.g. age-friendly cities and communities in their interventions aiming at improving the mobility of older people (World Health Organization, 2024).

### 4.2 Research and policy implications

This review summarises current literature on different interventions for mobility. Overall, the volume of experimental or quasi-experimental studies assessing intervention effects on mobility is limited, hence the need for better-quality studies with low risk of bias. Furthermore, evidence on interventions to improve mobility in age-friendly domains other than transport, housing and urban design/outdoor spaces are much needed (to a lesser extent for healthcare, see (Welch et al., 2021)) . There is also very limited evidence coming from low- and middle-income settings in all age-friendly domains.

Heterogeneity in operational definitions and the choice of mobility measures was one of the core challenges in attempting to summarise findings. To promote the standardisation of mobility measures across cities and communities worldwide, future research would benefit from mutually agreed upon definitions and conceptualisations of mobility, as well as more standardised measurements of mobility to promote data homogeneity developed with older people themselves, which would enable more robust evaluations and better reflect what matters to them in their daily activities (Conroy and Oppen, 2023).

Studies highlighted the effectiveness of at-home assistive technology interventions in mobility-related outcomes, slowing the decline of functional independence within frail older adult groups. Moreover, the review highlighted the effectiveness of interventions that reduced public transport fees for older age groups in improving overall mobility options, as well as how driving training increased the driving knowledge of older adults and reduced risky on-road behaviors. Interventions promoting ease and safety of usage, both for transport and in urban design, led to greater satisfaction. Policymakers should consider prioritising universal accessibility for both transport and urban spaces, rendering daily life more age-friendly, and thus leading to more service use and ultimately improved mobility. Policymakers should ensure age-friendly interventions are contextually sensitive to their built and social environments, in order to influence the specific domain that will most benefit the mobility and overall functional ability of older adults.

### 4.3 Review limitations

This review was limited by a few factors. Primarily, the potential for publication bias in this body of literature, where most studies found statistically significant effects for their evaluated interventions, means this review's findings should be interpreted with caution.

It is also possible that our search strategy could not fully capture all relevant studies due to the sheer volume of research on healthy ageing but also of research on transport, housing and urban development not directly aimed at older people. On the other hand, most evaluations were excluded from our review due to their study designs, despite the large body of literature initially identified, particularly because of the lack of a control group required to establish the causal effect of the intervention. For instance, despite its anticipated relevance, we did not identify studies assessing the accessibility of housing infrastructures using quasi-experimental or experimental designs.

This review could have further benefitted from a meta-analysis of its included studies. Because the records assessed mobility through a variety of study designs, interventions, and outcome measures, the overall presence of conceptual and methodological heterogeneity meant that a meta-analysis could not be performed.

Finally, as literature in this field is growing, our systematic review may not be capturing the latest experimental and quasi-experimental research on built-environment interventions and their impact on the mobility of older adults. For example, one study published after the end of our search evaluated the impact of a new metro line operation in Hong Kong found that this transportation infrastructural change increased metro usage, showing the potential to improve the subjective wellbeing of older adults (Sun and Du, 2023). Another recent study assessing the effects of a new bus rapid transit in El Paso, Texas, found that this intervention increased the mobility of older adults, measured by an increase in visits to local businesses (Song et al., 2023).

## 5 Conclusions

This systematic review, which evaluated the effectiveness of transportation, housing, and urban design interventions on the mobility and other related outcomes of city-dwelling older adults, yielded mixed results, but generally shows that such interventions in the social and built environment have the potential to produce a positive impact. Significant findings include the effectiveness of driving training in reducing risky on-road behaviors and improving driver knowledge, as well as the effectiveness of public transport interventions, such as bus fare reductions, in generating consistently positive outcomes on mobility. However, the current body of literature in this field is limited by its scarce volume of randomised control trials and quasi-experimental designs, making it difficult to establish if interventions have a causal effect on the mobility of older adults. Additionally, our review highlighted a notable heterogeneity across studies in how mobility was defined: future research would benefit from the use of a standardized definition of mobility to allow for better comparisons, as well as from the use of more rigorous methods to identify causal links.

## Disclaimer

The authors alone are responsible for the views expressed in this article and they do not necessarily represent the views, decisions or policies of the institutions with which they are affiliated.

This is an Open Access article published under the CC BY 3.0 IGO license which permits unrestricted use, distribution, and reproduction in any medium, provided the original work is properly cited. In any use of this article, there should be no suggestion that WHO endorses any specific organisation, products or services. The use of the WHO logo is not permitted. This notice should be preserved along with the article’s original URL.

## CRediT authorship contribution statement

**Thiago Hérick de Sá:** Writing – original draft, Validation, Methodology, Conceptualization. **Daniele Sudsataya:** Writing – review & editing, Writing – original draft. **Andra Fry:** Writing – review & editing, Methodology. **Nazak Salehi:** Data curation. **Aishwarya Katiki:** Data curation. **Megan Mcleod:** Data curation. **Greg Rathmell:** Data curation. **Jon Cylus:** Writing – review & editing. **Louise Lafortune:** Writing – review & editing. **Tine Buffel:** Writing – review & editing. **Patty Doran:** Writing – review & editing. **Alana Officer:** Writing – review & editing, Methodology, Conceptualization. **Huseyin Naci:** Writing – original draft, Validation, Methodology, Conceptualization.

## Declaration of competing interest

The authors declare that they have no known competing financial interests or personal relationships that could have appeared to influence the work reported in this paper.

## Figures and Tables

**Table:** 

1	(ageing or aging or elder* or eldest or geriatric* or gerontolog* or "later life” or "later in life” or "oldest old*" or pensioner* or senior* or senium or "very old*" or (old* adj3 (age* or adult* or citizen* or female or females or generation* or individual* or male or males or man or men or people* or person* or population* or woman or women or bicyclist* or cyclist* or driver* or motorcyclist* or motorist* or passenger* or pedestrian* or rider* or "road user*" or traveler* or walker*))).ti,ab,kf,kw.	1407804
2	(age? adj3 (over or older) adj2 (5# or 6# or 7# or 8# or 9#)).ti,ab,kf,kw.	50004
3	(“ ≥ 5# years old” or ">5# years old” or " ≥ 6# years old” or ">6# years old” or " ≥ 7# years old” or ">7# years old” or " ≥ 8# years old” or ">8# years old” or " ≥ 9# years old” or ">9# years old").ti,ab,kf,kw.	64777
4	1 or 2 or 3	1452488
5	(“functional abilit*" or "functional capacit*" or "functional health*" or "physical condition*" or "physical function*" or mobility or mobile or move or movement* or moving or "age* in place” or "aging in place” or "active life” or "active lives” or "active living” or ((active or healthy) adj3 (ageing or aging)) or (friendl* adj3 (age or ages or aged or aging or ageing or elder*))).ti,ab,kf,kw.	825848
6	(intervention* or effect* or evaluat* or impact* or controlled or "control group*" or (before adj5 after) or (pre adj5 post) or "pre-intervention*" or preintervention* or "post-intervention*" or postintervention* or "pre-post” or ((pretest* or "pre test*") and (posttest* or "post test*")) or quasiexperiment* or "quasi experiment*" or "pseudo experiment*" or pseudoexperiment* or "time series” or "time point*" or "repeated measur*").ti,ab,kf,kw.	12277191
7	(“active* transport*" or "active* travel*" or ((bicycl* or cycl* or driv* or motorcycl* or walk*) adj3 (coach* or course* or educat* or instruct* or lesson* or school* or train*)) or ((bicycl* or cycli* or driv* or motorcycl* or motorist* or passenger* or pedestrian* or rider* or walk* or traffic* or transport* or automobile* or bus* or car or cars or carpark* or "dial a ride” or garage* or metro or railway* or parking* or sidewalk* or subway* or taxi* or train or trains or underground or vehicle* or crossing* or highway* or lane or lanes or road or roads or street*) adj3 (abilit* or access* or afford* or behaviour* or behavior* or capacit* or danger* or difficult* or ergonomic* or habit* or safe* or stable or stability or unsafe or ((fare* or price* or ticket*) adj3 (discount* or free or reduc*)) or accident* or collision* or crash* or hazard* or incident* or speed*)) or ((bicycl* or cycli* or driv* or motorcycl* or automobile* or bus* or car or cars or metro or railway* or subway* or taxi* or train or trains or underground or vehicle*) adj3 (adapt* or alteration* or modif* or support or usability))).ti,ab,kf,kw.	154640
8	((ambient* adj3 assist* adj3 living) or ((smart or automat*) adj3 home*) or (smart adj3 environment*) or ((home* or hous* or residen* or accommodation* or bath* or bedroom* or kitchen* or room* or shower* or stair* or toilet*) adj3 (access* or adapt* or alteration* or modif* or renovat* or support or usability)) or ((elevator* or handrail* or "hand rail*" or lift or lifts or ramp or ramps or gerontechnolog* or "voice assistant*" or (service adj3 robot*) or ((assist* or intelligen* or safe* or "self-help*" or welfare*) adj3 (aid or aids or device* or platform* or robot* or solution* or technolog*))) and (home* or hous* or residen* or accommodation* or city or cities or communit* or environment* or neighb* or rural or societ* or town* or urban or village*))).ti,ab,kf,kw.	37630
9	(((urban or rural) adj3 (plan* or develop* or design*)) or ((built or green or neighb* or street* or rural or urban or cycling or driving or walking) adj3 environment*) or ((neighb* or rural or urban) adj3 space*) or amenit* or "communit* environment*" or greener* or greenness or greenspace* or greenway* or ((green or blue) adj3 (area* or space*)) or "land use*" or streetscape* or (street* adj3 connect*) or "rural renewal*" or "urban renewal*" or "rural form*" or "urban form*" or walkabilit* or ((population or residential or retail or rural or urban) adj3 densit*) or ((aesthetic* or garden or gardens or park or parks or ((plan* or develop* or design*) adj3 (facility or facilities)) or ((outdoor* or public) adj3 (space* or building* or facility or facilities*)) or ((indoor* or living or natural or objective or outdoor* or perceived or physical) adj3 environment*)) and (home* or hous* or residen* or accommodation* or city or cities or communit* or neighb* or rural or societ* or town* or urban or village*)) or ((neighb* adj3 (space* or building* or facility or facilities)) and (home* or hous* or residen* or accommodation* or city or cities or communit* or rural or societ* or town* or urban or village*)) or ((communit* adj3 (space* or building* or facility or facilities)) and (home* or hous* or residen* or accommodation* or city or cities or neighb* or rural or societ* or town* or urban or village*)) or (infrastructure* and (city or cities or communit* or cycling or driving or neighb* or pedestrian or public or rural or town* or transport* or urban or village*))).ti,ab,kf,kw.	124915
10	7 or 8 or 9	309685
11	4 and 5 and 6 and 10	3591
12	exp animals/not humans/	5022910
13	11 not 12	3565
14	(comment or editorial or letter).pt.	2073228
15	13 not 14	3556

**Table:** 

1	(ageing or aging or elder* or eldest or geriatric* or gerontolog* or "later life” or "later in life” or "oldest old*" or pensioner* or senior* or senium or "very old*" or (old* adj3 (age* or adult* or citizen* or female or females or generation* or individual* or male or males or man or men or people* or person* or population* or woman or women or bicyclist* or cyclist* or driver* or motorcyclist* or motorist* or passenger* or pedestrian* or rider* or "road user*" or traveler* or walker*))).ti,ab,kf,kw.	1952440
2	(age? adj3 (over or older) adj2 (5# or 6# or 7# or 8# or 9#)).ti,ab,kf,kw.	74594
3	(“ ≥ 5# years old” or ">5# years old” or " ≥ 6# years old” or ">6# years old” or " ≥ 7# years old” or ">7# years old” or " ≥ 8# years old” or ">8# years old” or " ≥ 9# years old” or ">9# years old").ti,ab,kf,kw.	130187
4	1 or 2 or 3	2035702
5	(“functional abilit*" or "functional capacit*" or "functional health*" or "physical condition*" or "physical function*" or mobility or mobile or move or movement* or moving or "age* in place” or "aging in place” or "active life” or "active lives” or "active living” or ((active or healthy) adj3 (ageing or aging)) or (friendl* adj3 (age or ages or aged or aging or ageing or elder*))).ti,ab,kf,kw.	1028650
6	(intervention* or effect* or evaluat* or impact* or controlled or "control group*" or (before adj5 after) or (pre adj5 post) or "pre-intervention*" or preintervention* or "post-intervention*" or postintervention* or "pre-post” or ((pretest* or "pre test*") and (posttest* or "post test*")) or quasiexperiment* or "quasi experiment*" or "pseudo experiment*" or pseudoexperiment* or "time series” or "time point*" or "repeated measur*").ti,ab,kf,kw.	15577161
7	(“active* transport*" or "active* travel*" or ((bicycl* or cycl* or driv* or motorcycl* or walk*) adj3 (coach* or course* or educat* or instruct* or lesson* or school* or train*)) or ((bicycl* or cycli* or driv* or motorcycl* or motorist* or passenger* or pedestrian* or rider* or walk* or traffic* or transport* or automobile* or bus* or car or cars or carpark* or "dial a ride” or garage* or metro or railway* or parking* or sidewalk* or subway* or taxi* or train or trains or underground or vehicle* or crossing* or highway* or lane or lanes or road or roads or street*) adj3 (abilit* or access* or afford* or behaviour* or behavior* or capacit* or danger* or difficult* or ergonomic* or habit* or safe* or stable or stability or unsafe or ((fare* or price* or ticket*) adj3 (discount* or free or reduc*)) or accident* or collision* or crash* or hazard* or incident* or speed*)) or ((bicycl* or cycli* or driv* or motorcycl* or automobile* or bus* or car or cars or metro or railway* or subway* or taxi* or train or trains or underground or vehicle*) adj3 (adapt* or alteration* or modif* or support or usability))).ti,ab,kf,kw.	189289
8	((ambient* adj3 assist* adj3 living) or ((smart or automat*) adj3 home*) or (smart adj3 environment*) or ((home* or hous* or residen* or accommodation* or bath* or bedroom* or kitchen* or room* or shower* or stair* or toilet*) adj3 (access* or adapt* or alteration* or modif* or renovat* or support or usability)) or ((elevator* or handrail* or "hand rail*" or lift or lifts or ramp or ramps or gerontechnolog* or "voice assistant*" or (service adj3 robot*) or ((assist* or intelligen* or safe* or "self-help*" or welfare*) adj3 (aid or aids or device* or platform* or robot* or solution* or technolog*))) and (home* or hous* or residen* or accommodation* or city or cities or communit* or environment* or neighb* or rural or societ* or town* or urban or village*))).ti,ab,kf,kw.	56212
9	(((urban or rural) adj3 (plan* or develop* or design*)) or ((built or green or neighb* or street* or rural or urban or cycling or driving or walking) adj3 environment*) or ((neighb* or rural or urban) adj3 space*) or amenit* or "communit* environment*" or greener* or greenness or greenspace* or greenway* or ((green or blue) adj3 (area* or space*)) or "land use*" or streetscape* or (street* adj3 connect*) or "rural renewal*" or "urban renewal*" or "rural form*" or "urban form*" or walkabilit* or ((population or residential or retail or rural or urban) adj3 densit*) or ((aesthetic* or garden or gardens or park or parks or ((plan* or develop* or design*) adj3 (facility or facilities)) or ((outdoor* or public) adj3 (space* or building* or facility or facilities*)) or ((indoor* or living or natural or objective or outdoor* or perceived or physical) adj3 environment*)) and (home* or hous* or residen* or accommodation* or city or cities or communit* or neighb* or rural or societ* or town* or urban or village*)) or ((neighb* adj3 (space* or building* or facility or facilities)) and (home* or hous* or residen* or accommodation* or city or cities or communit* or rural or societ* or town* or urban or village*)) or ((communit* adj3 (space* or building* or facility or facilities)) and (home* or hous* or residen* or accommodation* or city or cities or neighb* or rural or societ* or town* or urban or village*)) or (infrastructure* and (city or cities or communit* or cycling or driving or neighb* or pedestrian or public or rural or town* or transport* or urban or village*))).ti,ab,kf,kw.	147736
10	7 or 8 or 9	384130
11	4 and 5 and 6 and 10	5044
12	exp animal/or exp animal experiment/or nonhuman/	30722964
13	exp human/or exp human experiment/	23780248
14	12 not 13	6943905
15	11 not 14	4985
16	(editorial or letter).pt.	1960913
17	15 not 16	4980

**Table:** 

1	(ageing or aging or elder* or eldest or geriatric* or gerontolog* or "later life” or "later in life” or "oldest old*" or pensioner* or senior* or senium or "very old*" or (old* adj3 (age* or adult* or citizen* or female or females or generation* or individual* or male or males or man or men or people* or person* or population* or woman or women or bicyclist* or cyclist* or driver* or motorcyclist* or motorist* or passenger* or pedestrian* or rider* or "road user*" or traveler* or walker*))).ti,ab,id.	350117
2	(age? adj3 (over or older) adj2 (5# or 6# or 7# or 8# or 9#)).ti,ab,id.	16000
3	(“ ≥ 5# years old” or ">5# years old” or " ≥ 6# years old” or ">6# years old” or " ≥ 7# years old” or ">7# years old” or " ≥ 8# years old” or ">8# years old” or " ≥ 9# years old” or ">9# years old").ti,ab,id.	7644
4	1 or 2 or 3	355354
5	(“functional abilit*" or "functional capacit*" or "functional health*" or "physical condition*" or "physical function*" or mobility or mobile or move or movement* or moving or "age* in place” or "aging in place” or "active life” or "active lives” or "active living” or ((active or healthy) adj3 (ageing or aging)) or (friendl* adj3 (age or ages or aged or aging or ageing or elder*))).ti,ab,id.	272558
6	(intervention* or effect* or evaluat* or impact* or controlled or "control group*" or (before adj5 after) or (pre adj5 post) or "pre-intervention*" or preintervention* or "post-intervention*" or postintervention* or "pre-post” or ((pretest* or "pre test*") and (posttest* or "post test*")) or quasiexperiment* or "quasi experiment*" or "pseudo experiment*" or pseudoexperiment* or "time series” or "time point*" or "repeated measur*").ti,ab,id.	2372842
7	(“active* transport*" or "active* travel*" or ((bicycl* or cycl* or driv* or motorcycl* or walk*) adj3 (coach* or course* or educat* or instruct* or lesson* or school* or train*)) or ((bicycl* or cycli* or driv* or motorcycl* or motorist* or passenger* or pedestrian* or rider* or walk* or traffic* or transport* or automobile* or bus* or car or cars or carpark* or "dial a ride” or garage* or metro or railway* or parking* or sidewalk* or subway* or taxi* or train or trains or underground or vehicle* or crossing* or highway* or lane or lanes or road or roads or street*) adj3 (abilit* or access* or afford* or behaviour* or behavior* or capacit* or danger* or difficult* or ergonomic* or habit* or safe* or stable or stability or unsafe or ((fare* or price* or ticket*) adj3 (discount* or free or reduc*)) or accident* or collision* or crash* or hazard* or incident* or speed*)) or ((bicycl* or cycli* or driv* or motorcycl* or automobile* or bus* or car or cars or metro or railway* or subway* or taxi* or train or trains or underground or vehicle*) adj3 (adapt* or alteration* or modif* or support or usability))).ti,ab,id.	42907
8	((ambient* adj3 assist* adj3 living) or ((smart or automat*) adj3 home*) or (smart adj3 environment*) or ((home* or hous* or residen* or accommodation* or bath* or bedroom* or kitchen* or room* or shower* or stair* or toilet*) adj3 (access* or adapt* or alteration* or modif* or renovat* or support or usability)) or ((elevator* or handrail* or "hand rail*" or lift or lifts or ramp or ramps or gerontechnolog* or "voice assistant*" or (service adj3 robot*) or ((assist* or intelligen* or safe* or "self-help*" or welfare*) adj3 (aid or aids or device* or platform* or robot* or solution* or technolog*))) and (home* or hous* or residen* or accommodation* or city or cities or communit* or environment* or neighb* or rural or societ* or town* or urban or village*))).ti,ab,id.	13552
9	(((urban or rural) adj3 (plan* or develop* or design*)) or ((built or green or neighb* or street* or rural or urban or cycling or driving or walking) adj3 environment*) or ((neighb* or rural or urban) adj3 space*) or amenit* or "communit* environment*" or greener* or greenness or greenspace* or greenway* or ((green or blue) adj3 (area* or space*)) or "land use*" or streetscape* or (street* adj3 connect*) or "rural renewal*" or "urban renewal*" or "rural form*" or "urban form*" or walkabilit* or ((population or residential or retail or rural or urban) adj3 densit*) or ((aesthetic* or garden or gardens or park or parks or ((plan* or develop* or design*) adj3 (facility or facilities)) or ((outdoor* or public) adj3 (space* or building* or facility or facilities*)) or ((indoor* or living or natural or objective or outdoor* or perceived or physical) adj3 environment*)) and (home* or hous* or residen* or accommodation* or city or cities or communit* or neighb* or rural or societ* or town* or urban or village*)) or ((neighb* adj3 (space* or building* or facility or facilities)) and (home* or hous* or residen* or accommodation* or city or cities or communit* or rural or societ* or town* or urban or village*)) or ((communit* adj3 (space* or building* or facility or facilities)) and (home* or hous* or residen* or accommodation* or city or cities or neighb* or rural or societ* or town* or urban or village*)) or (infrastructure* and (city or cities or communit* or cycling or driving or neighb* or pedestrian or public or rural or town* or transport* or urban or village*))).ti,ab,id.	41402
10	7 or 8 or 9	94374
11	4 and 5 and 6 and 10	1379
12	(animal not human).po.	378204
13	11 not 12	1373

**Table:** 

S1	TI (ageing or aging or elder* or eldest or geriatric* or gerontolog* or "later life” or "later in life” or "oldest old*" or pensioner* or senior* or senium or "very old*" or (old* N3 (age* or adult* or citizen* or female or females or generation* or individual* or male or males or man or men or people* or person* or population* or woman or women or bicyclist* or cyclist* or driver* or motorcyclist* or motorist* or passenger* or pedestrian* or rider* or "road user*" or traveler* or walker*)) or (age# N3 (over or older) N2 (5? or 6? or 7? or 8? or 9?)) or (“ ≥ 5? years old” or ">5? years old” or " ≥ 6? years old” or ">6? years old” or " ≥ 7? years old” or ">7? years old” or " ≥ 8? years old” or ">8? years old” or " ≥ 9? years old” or ">9? years old”)) OR AB (ageing or aging or elder* or eldest or geriatric* or gerontolog* or "later life” or "later in life” or "oldest old*" or pensioner* or senior* or senium or "very old*" or (old* N3 (age* or adult* or citizen* or female or females or generation* or individual* or male or males or man or men or people* or person* or population* or woman or women or bicyclist* or cyclist* or driver* or motorcyclist* or motorist* or passenger* or pedestrian* or rider* or "road user*" or traveler* or walker*)) or (age# N3 (over or older) N2 (5? or 6? or 7? or 8? or 9?)) or (“ ≥ 5? years old” or ">5? years old” or " ≥ 6? years old” or ">6? years old” or " ≥ 7? years old” or ">7? years old” or " ≥ 8? years old” or ">8? years old” or " ≥ 9? years old” or ">9? years old”))	
S2	TI (“functional abilit*" or "functional capacit*" or "functional health*" or "physical condition*" or "physical function*" or mobility or mobile or move or movement* or moving or "age* in place” or "aging in place” or "active life” or "active lives” or "active living” or ((active or healthy) N3 (ageing or aging)) or (friendl* N3 (age or ages or aged or aging or ageing or elder*))) OR AB (“functional abilit*" or "functional capacit*" or "functional health*" or "physical condition*" or "physical function*" or mobility or mobile or move or movement* or moving or "age* in place” or "aging in place” or "active life” or "active lives” or "active living” or ((active or healthy) N3 (ageing or aging)) or (friendl* N3 (age or ages or aged or aging or ageing or elder*)))	
S3	TI (intervention* or effect* or evaluat* or impact* or controlled or "control group*" or (before N5 after) or (pre N5 post) or "pre-intervention*" or preintervention* or "post-intervention*" or postintervention* or "pre-post” or ((pretest* or "pre test*") and (posttest* or "post test*")) or quasiexperiment* or "quasi experiment*" or "pseudo experiment*" or pseudoexperiment* or "time series” or "time point*" or "repeated measur*") OR AB (intervention* or effect* or evaluat* or impact* or controlled or "control group*" or (before N5 after) or (pre N5 post) or "pre-intervention*" or preintervention* or "post-intervention*" or postintervention* or "pre-post” or ((pretest* or "pre test*") and (posttest* or "post test*")) or quasiexperiment* or "quasi experiment*" or "pseudo experiment*" or pseudoexperiment* or "time series” or "time point*" or "repeated measur*")	
S4	TI (“active* transport*" or "active* travel*" or ((bicycl* or cycl* or driv* or motorcycl* or walk*) N3 (coach* or course* or educat* or instruct* or lesson* or school* or train*)) or ((bicycl* or cycli* or driv* or motorcycl* or motorist* or passenger* or pedestrian* or rider* or walk* or traffic* or transport* or automobile* or bus* or car or cars or carpark* or "dial a ride” or garage* or metro or railway* or parking* or sidewalk* or subway* or taxi* or train or trains or underground or vehicle* or crossing* or highway* or lane or lanes or road or roads or street*) N3 (abilit* or access* or afford* or behaviour* or behavior* or capacit* or danger* or difficult* or ergonomic* or habit* or safe* or stable or stability or unsafe or ((fare* or price* or ticket*) N3 (discount* or free or reduc*)) or accident* or collision* or crash* or hazard* or incident* or speed*)) or ((bicycl* or cycli* or driv* or motorcycl* or automobile* or bus* or car or cars or metro or railway* or subway* or taxi* or train or trains or underground or vehicle*) N3 (adapt* or alteration* or modif* or support or usability))) OR AB (“active* transport*" or "active* travel*" or ((bicycl* or cycl* or driv* or motorcycl* or walk*) N3 (coach* or course* or educat* or instruct* or lesson* or school* or train*)) or ((bicycl* or cycli* or driv* or motorcycl* or motorist* or passenger* or pedestrian* or rider* or walk* or traffic* or transport* or automobile* or bus* or car or cars or carpark* or "dial a ride” or garage* or metro or railway* or parking* or sidewalk* or subway* or taxi* or train or trains or underground or vehicle* or crossing* or highway* or lane or lanes or road or roads or street*) N3 (abilit* or access* or afford* or behaviour* or behavior* or capacit* or danger* or difficult* or ergonomic* or habit* or safe* or stable or stability or unsafe or ((fare* or price* or ticket*) N3 (discount* or free or reduc*)) or accident* or collision* or crash* or hazard* or incident* or speed*)) or ((bicycl* or cycli* or driv* or motorcycl* or automobile* or bus* or car or cars or metro or railway* or subway* or taxi* or train or trains or underground or vehicle*) N3 (adapt* or alteration* or modif* or support or usability)))	
S5	TI ((ambient* N3 assist* N3 living) or ((smart or automat*) N3 home*) or (smart N3 environment*) or ((home* or hous* or residen* or accommodation* or bath* or bedroom* or kitchen* or room* or shower* or stair* or toilet*) N3 (access* or adapt* or alteration* or modif* or renovat* or support or usability)) or ((elevator* or handrail* or "hand rail*" or lift or lifts or ramp or ramps or gerontechnolog* or "voice assistant*" or (service N3 robot*) or ((assist* or intelligen* or safe* or "self-help*" or welfare*) N3 (aid or aids or device* or platform* or robot* or solution* or technolog*))) and (home* or hous* or residen* or accommodation* or city or cities or communit* or environment* or neighb* or rural or societ* or town* or urban or village*))) OR AB ((ambient* N3 assist* N3 living) or ((smart or automat*) N3 home*) or (smart N3 environment*) or ((home* or hous* or residen* or accommodation* or bath* or bedroom* or kitchen* or room* or shower* or stair* or toilet*) N3 (access* or adapt* or alteration* or modif* or renovat* or support or usability)) or ((elevator* or handrail* or "hand rail*" or lift or lifts or ramp or ramps or gerontechnolog* or "voice assistant*" or (service N3 robot*) or ((assist* or intelligen* or safe* or "self-help*" or welfare*) N3 (aid or aids or device* or platform* or robot* or solution* or technolog*))) and (home* or hous* or residen* or accommodation* or city or cities or communit* or environment* or neighb* or rural or societ* or town* or urban or village*)))	
S6	TI (((urban or rural) N3 (plan* or develop* or design*)) or ((built or green or neighb* or street* or rural or urban or cycling or driving or walking) N3 environment*) or ((neighb* or rural or urban) N3 space*) or amenit* or "communit* environment*" or greener* or greenness or greenspace* or greenway* or ((green or blue) N3 (area* or space*)) or "land use*" or streetscape* or (street* N3 connect*) or "rural renewal*" or "urban renewal*" or "rural form*" or "urban form*" or walkabilit* or ((population or residential or retail or rural or urban) N3 densit*) or ((aesthetic* or garden or gardens or park or parks or ((plan* or develop* or design*) N3 (facility or facilities)) or ((outdoor* or public) N3 (space* or building* or facility or facilities*)) or ((indoor* or living or natural or objective or outdoor* or perceived or physical) N3 environment*)) and (home* or hous* or residen* or accommodation* or city or cities or communit* or neighb* or rural or societ* or town* or urban or village*)) or ((neighb* N3 (space* or building* or facility or facilities)) and (home* or hous* or residen* or accommodation* or city or cities or communit* or rural or societ* or town* or urban or village*)) or ((communit* N3 (space* or building* or facility or facilities)) and (home* or hous* or residen* or accommodation* or city or cities or neighb* or rural or societ* or town* or urban or village*)) or (infrastructure* and (city or cities or communit* or cycling or driving or neighb* or pedestrian or public or rural or town* or transport* or urban or village*))) OR AB (((urban or rural) N3 (plan* or develop* or design*)) or ((built or green or neighb* or street* or rural or urban or cycling or driving or walking) N3 environment*) or ((neighb* or rural or urban) N3 space*) or amenit* or "communit* environment*" or greener* or greenness or greenspace* or greenway* or ((green or blue) N3 (area* or space*)) or "land use*" or streetscape* or (street* N3 connect*) or "rural renewal*" or "urban renewal*" or "rural form*" or "urban form*" or walkabilit* or ((population or residential or retail or rural or urban) N3 densit*) or ((aesthetic* or garden or gardens or park or parks or ((plan* or develop* or design*) N3 (facility or facilities)) or ((outdoor* or public) N3 (space* or building* or facility or facilities*)) or ((indoor* or living or natural or objective or outdoor* or perceived or physical) N3 environment*)) and (home* or hous* or residen* or accommodation* or city or cities or communit* or neighb* or rural or societ* or town* or urban or village*)) or ((neighb* N3 (space* or building* or facility or facilities)) and (home* or hous* or residen* or accommodation* or city or cities or communit* or rural or societ* or town* or urban or village*)) or ((communit* N3 (space* or building* or facility or facilities)) and (home* or hous* or residen* or accommodation* or city or cities or neighb* or rural or societ* or town* or urban or village*)) or (infrastructure* and (city or cities or communit* or cycling or driving or neighb* or pedestrian or public or rural or town* or transport* or urban or village*)))	
S7	S4 OR S5 OR S6	
S8	S1 AND S2 AND S3 AND S7	
S9	(MH "Animals+" OR MH "Animal Studies” OR TI animal model*) NOT MH "Human”	
S10	S8 NOT S9	2047

**Table:** 

S1	TI (ageing or aging or elder* or eldest or geriatric* or gerontolog* or "later life” or "later in life” or "oldest old*" or pensioner* or senior* or senium or "very old*" or (old* N3 (age* or adult* or citizen* or female or females or generation* or individual* or male or males or man or men or people* or person* or population* or woman or women or bicyclist* or cyclist* or driver* or motorcyclist* or motorist* or passenger* or pedestrian* or rider* or "road user*" or traveler* or walker*)) or (age# N3 (over or older) N2 (5? or 6? or 7? or 8? or 9?)) or (“ ≥ 5? years old” or ">5? years old” or " ≥ 6? years old” or ">6? years old” or " ≥ 7? years old” or ">7? years old” or " ≥ 8? years old” or ">8? years old” or " ≥ 9? years old” or ">9? years old”)) OR AB (ageing or aging or elder* or eldest or geriatric* or gerontolog* or "later life” or "later in life” or "oldest old*" or pensioner* or senior* or senium or "very old*" or (old* N3 (age* or adult* or citizen* or female or females or generation* or individual* or male or males or man or men or people* or person* or population* or woman or women or bicyclist* or cyclist* or driver* or motorcyclist* or motorist* or passenger* or pedestrian* or rider* or "road user*" or traveler* or walker*)) or (age# N3 (over or older) N2 (5? or 6? or 7? or 8? or 9?)) or (“ ≥ 5? years old” or ">5? years old” or " ≥ 6? years old” or ">6? years old” or " ≥ 7? years old” or ">7? years old” or " ≥ 8? years old” or ">8? years old” or " ≥ 9? years old” or ">9? years old”)) OR KW (ageing or aging or elder* or eldest or geriatric* or gerontolog* or "later life” or "later in life” or "oldest old*" or pensioner* or senior* or senium or "very old*" or (old* N3 (age* or adult* or citizen* or female or females or generation* or individual* or male or males or man or men or people* or person* or population* or woman or women or bicyclist* or cyclist* or driver* or motorcyclist* or motorist* or passenger* or pedestrian* or rider* or "road user*" or traveler* or walker*)) or (age# N3 (over or older) N2 (5? or 6? or 7? or 8? or 9?)) or (“ ≥ 5? years old” or ">5? years old” or " ≥ 6? years old” or ">6? years old” or " ≥ 7? years old” or ">7? years old” or " ≥ 8? years old” or ">8? years old” or " ≥ 9? years old” or ">9? years old”))	
S2	TI (“functional abilit*" or "functional capacit*" or "functional health*" or "physical condition*" or "physical function*" or mobility or mobile or move or movement* or moving or "age* in place” or "aging in place” or "active life” or "active lives” or "active living” or ((active or healthy) N3 (ageing or aging)) or (friendl* N3 (age or ages or aged or aging or ageing or elder*))) OR AB (“functional abilit*" or "functional capacit*" or "functional health*" or "physical condition*" or "physical function*" or mobility or mobile or move or movement* or moving or "age* in place” or "aging in place” or "active life” or "active lives” or "active living” or ((active or healthy) N3 (ageing or aging)) or (friendl* N3 (age or ages or aged or aging or ageing or elder*))) OR KW (“functional abilit*" or "functional capacit*" or "functional health*" or "physical condition*" or "physical function*" or mobility or mobile or move or movement* or moving or "age* in place” or "aging in place” or "active life” or "active lives” or "active living” or ((active or healthy) N3 (ageing or aging)) or (friendl* N3 (age or ages or aged or aging or ageing or elder*)))	
S3	TI (intervention* or effect* or evaluat* or impact* or controlled or "control group*" or (before N5 after) or (pre N5 post) or "pre-intervention*" or preintervention* or "post-intervention*" or postintervention* or "pre-post” or ((pretest* or "pre test*") and (posttest* or "post test*")) or quasiexperiment* or "quasi experiment*" or "pseudo experiment*" or pseudoexperiment* or "time series” or "time point*" or "repeated measur*") OR AB (intervention* or effect* or evaluat* or impact* or controlled or "control group*" or (before N5 after) or (pre N5 post) or "pre-intervention*" or preintervention* or "post-intervention*" or postintervention* or "pre-post” or ((pretest* or "pre test*") and (posttest* or "post test*")) or quasiexperiment* or "quasi experiment*" or "pseudo experiment*" or pseudoexperiment* or "time series” or "time point*" or "repeated measur*") OR KW (intervention* or effect* or evaluat* or impact* or controlled or "control group*" or (before N5 after) or (pre N5 post) or "pre-intervention*" or preintervention* or "post-intervention*" or postintervention* or "pre-post” or ((pretest* or "pre test*") and (posttest* or "post test*")) or quasiexperiment* or "quasi experiment*" or "pseudo experiment*" or pseudoexperiment* or "time series” or "time point*" or "repeated measur*")	
S4	TI (“active* transport*" or "active* travel*" or ((bicycl* or cycl* or driv* or motorcycl* or walk*) N3 (coach* or course* or educat* or instruct* or lesson* or school* or train*)) or ((bicycl* or cycli* or driv* or motorcycl* or motorist* or passenger* or pedestrian* or rider* or walk* or traffic* or transport* or automobile* or bus* or car or cars or carpark* or "dial a ride” or garage* or metro or railway* or parking* or sidewalk* or subway* or taxi* or train or trains or underground or vehicle* or crossing* or highway* or lane or lanes or road or roads or street*) N3 (abilit* or access* or afford* or behaviour* or behavior* or capacit* or danger* or difficult* or ergonomic* or habit* or safe* or stable or stability or unsafe or ((fare* or price* or ticket*) N3 (discount* or free or reduc*)) or accident* or collision* or crash* or hazard* or incident* or speed*)) or ((bicycl* or cycli* or driv* or motorcycl* or automobile* or bus* or car or cars or metro or railway* or subway* or taxi* or train or trains or underground or vehicle*) N3 (adapt* or alteration* or modif* or support or usability))) OR AB (“active* transport*" or "active* travel*" or ((bicycl* or cycl* or driv* or motorcycl* or walk*) N3 (coach* or course* or educat* or instruct* or lesson* or school* or train*)) or ((bicycl* or cycli* or driv* or motorcycl* or motorist* or passenger* or pedestrian* or rider* or walk* or traffic* or transport* or automobile* or bus* or car or cars or carpark* or "dial a ride” or garage* or metro or railway* or parking* or sidewalk* or subway* or taxi* or train or trains or underground or vehicle* or crossing* or highway* or lane or lanes or road or roads or street*) N3 (abilit* or access* or afford* or behaviour* or behavior* or capacit* or danger* or difficult* or ergonomic* or habit* or safe* or stable or stability or unsafe or ((fare* or price* or ticket*) N3 (discount* or free or reduc*)) or accident* or collision* or crash* or hazard* or incident* or speed*)) or ((bicycl* or cycli* or driv* or motorcycl* or automobile* or bus* or car or cars or metro or railway* or subway* or taxi* or train or trains or underground or vehicle*) N3 (adapt* or alteration* or modif* or support or usability))) OR KW (“active* transport*" or "active* travel*" or ((bicycl* or cycl* or driv* or motorcycl* or walk*) N3 (coach* or course* or educat* or instruct* or lesson* or school* or train*)) or ((bicycl* or cycli* or driv* or motorcycl* or motorist* or passenger* or pedestrian* or rider* or walk* or traffic* or transport* or automobile* or bus* or car or cars or carpark* or "dial a ride” or garage* or metro or railway* or parking* or sidewalk* or subway* or taxi* or train or trains or underground or vehicle* or crossing* or highway* or lane or lanes or road or roads or street*) N3 (abilit* or access* or afford* or behaviour* or behavior* or capacit* or danger* or difficult* or ergonomic* or habit* or safe* or stable or stability or unsafe or ((fare* or price* or ticket*) N3 (discount* or free or reduc*)) or accident* or collision* or crash* or hazard* or incident* or speed*)) or ((bicycl* or cycli* or driv* or motorcycl* or automobile* or bus* or car or cars or metro or railway* or subway* or taxi* or train or trains or underground or vehicle*) N3 (adapt* or alteration* or modif* or support or usability)))	
S5	TI ((ambient* N3 assist* N3 living) or ((smart or automat*) N3 home*) or (smart N3 environment*) or ((home* or hous* or residen* or accommodation* or bath* or bedroom* or kitchen* or room* or shower* or stair* or toilet*) N3 (access* or adapt* or alteration* or modif* or renovat* or support or usability)) or ((elevator* or handrail* or "hand rail*" or lift or lifts or ramp or ramps or gerontechnolog* or "voice assistant*" or (service N3 robot*) or ((assist* or intelligen* or safe* or "self-help*" or welfare*) N3 (aid or aids or device* or platform* or robot* or solution* or technolog*))) and (home* or hous* or residen* or accommodation* or city or cities or communit* or environment* or neighb* or rural or societ* or town* or urban or village*))) OR AB ((ambient* N3 assist* N3 living) or ((smart or automat*) N3 home*) or (smart N3 environment*) or ((home* or hous* or residen* or accommodation* or bath* or bedroom* or kitchen* or room* or shower* or stair* or toilet*) N3 (access* or adapt* or alteration* or modif* or renovat* or support or usability)) or ((elevator* or handrail* or "hand rail*" or lift or lifts or ramp or ramps or gerontechnolog* or "voice assistant*" or (service N3 robot*) or ((assist* or intelligen* or safe* or "self-help*" or welfare*) N3 (aid or aids or device* or platform* or robot* or solution* or technolog*))) and (home* or hous* or residen* or accommodation* or city or cities or communit* or environment* or neighb* or rural or societ* or town* or urban or village*))) OR KW ((ambient* N3 assist* N3 living) or ((smart or automat*) N3 home*) or (smart N3 environment*) or ((home* or hous* or residen* or accommodation* or bath* or bedroom* or kitchen* or room* or shower* or stair* or toilet*) N3 (access* or adapt* or alteration* or modif* or renovat* or support or usability)) or ((elevator* or handrail* or "hand rail*" or lift or lifts or ramp or ramps or gerontechnolog* or "voice assistant*" or (service N3 robot*) or ((assist* or intelligen* or safe* or "self-help*" or welfare*) N3 (aid or aids or device* or platform* or robot* or solution* or technolog*))) and (home* or hous* or residen* or accommodation* or city or cities or communit* or environment* or neighb* or rural or societ* or town* or urban or village*)))	
S6	TI (((urban or rural) N3 (plan* or develop* or design*)) or ((built or green or neighb* or street* or rural or urban or cycling or driving or walking) N3 environment*) or ((neighb* or rural or urban) N3 space*) or amenit* or "communit* environment*" or greener* or greenness or greenspace* or greenway* or ((green or blue) N3 (area* or space*)) or "land use*" or streetscape* or (street* N3 connect*) or "rural renewal*" or "urban renewal*" or "rural form*" or "urban form*" or walkabilit* or ((population or residential or retail or rural or urban) N3 densit*) or ((aesthetic* or garden or gardens or park or parks or ((plan* or develop* or design*) N3 (facility or facilities)) or ((outdoor* or public) N3 (space* or building* or facility or facilities*)) or ((indoor* or living or natural or objective or outdoor* or perceived or physical) N3 environment*)) and (home* or hous* or residen* or accommodation* or city or cities or communit* or neighb* or rural or societ* or town* or urban or village*)) or ((neighb* N3 (space* or building* or facility or facilities)) and (home* or hous* or residen* or accommodation* or city or cities or communit* or rural or societ* or town* or urban or village*)) or ((communit* N3 (space* or building* or facility or facilities)) and (home* or hous* or residen* or accommodation* or city or cities or neighb* or rural or societ* or town* or urban or village*)) or (infrastructure* and (city or cities or communit* or cycling or driving or neighb* or pedestrian or public or rural or town* or transport* or urban or village*))) OR AB (((urban or rural) N3 (plan* or develop* or design*)) or ((built or green or neighb* or street* or rural or urban or cycling or driving or walking) N3 environment*) or ((neighb* or rural or urban) N3 space*) or amenit* or "communit* environment*" or greener* or greenness or greenspace* or greenway* or ((green or blue) N3 (area* or space*)) or "land use*" or streetscape* or (street* N3 connect*) or "rural renewal*" or "urban renewal*" or "rural form*" or "urban form*" or walkabilit* or ((population or residential or retail or rural or urban) N3 densit*) or ((aesthetic* or garden or gardens or park or parks or ((plan* or develop* or design*) N3 (facility or facilities)) or ((outdoor* or public) N3 (space* or building* or facility or facilities*)) or ((indoor* or living or natural or objective or outdoor* or perceived or physical) N3 environment*)) and (home* or hous* or residen* or accommodation* or city or cities or communit* or neighb* or rural or societ* or town* or urban or village*)) or ((neighb* N3 (space* or building* or facility or facilities)) and (home* or hous* or residen* or accommodation* or city or cities or communit* or rural or societ* or town* or urban or village*)) or ((communit* N3 (space* or building* or facility or facilities)) and (home* or hous* or residen* or accommodation* or city or cities or neighb* or rural or societ* or town* or urban or village*)) or (infrastructure* and (city or cities or communit* or cycling or driving or neighb* or pedestrian or public or rural or town* or transport* or urban or village*))) OR KW (((urban or rural) N3 (plan* or develop* or design*)) or ((built or green or neighb* or street* or rural or urban or cycling or driving or walking) N3 environment*) or ((neighb* or rural or urban) N3 space*) or amenit* or "communit* environment*" or greener* or greenness or greenspace* or greenway* or ((green or blue) N3 (area* or space*)) or "land use*" or streetscape* or (street* N3 connect*) or "rural renewal*" or "urban renewal*" or "rural form*" or "urban form*" or walkabilit* or ((population or residential or retail or rural or urban) N3 densit*) or ((aesthetic* or garden or gardens or park or parks or ((plan* or develop* or design*) N3 (facility or facilities)) or ((outdoor* or public) N3 (space* or building* or facility or facilities*)) or ((indoor* or living or natural or objective or outdoor* or perceived or physical) N3 environment*)) and (home* or hous* or residen* or accommodation* or city or cities or communit* or neighb* or rural or societ* or town* or urban or village*)) or ((neighb* N3 (space* or building* or facility or facilities)) and (home* or hous* or residen* or accommodation* or city or cities or communit* or rural or societ* or town* or urban or village*)) or ((communit* N3 (space* or building* or facility or facilities)) and (home* or hous* or residen* or accommodation* or city or cities or neighb* or rural or societ* or town* or urban or village*)) or (infrastructure* and (city or cities or communit* or cycling or driving or neighb* or pedestrian or public or rural or town* or transport* or urban or village*)))	
S7	S5 OR S6 OR S7	
S8	S1 AND S2 AND S3 AND S7	540

**Table:** 

TITLE-ABS((ageing or aging or elder* or eldest or geriatric* or gerontolog* or "later life” or "later in life” or "oldest old*" or pensioner* or senior* or senium or "very old*" or (old* W/2 (age* or adult* or citizen* or female or females or generation* or individual* or male or males or man or men or people* or person* or population* or woman or women or bicyclist* or cyclist* or driver* or motorcyclist* or motorist* or passenger* or pedestrian* or rider* or "road user*" or traveler* or walker*))) AND (“functional abilit*" or "functional capacit*" or "functional health*" or "physical condition*" or "physical function*" or mobility or mobile or move or movement* or moving or "age* in place” or "aging in place” or "active life” or "active lives” or "active living” or ((active or healthy) W/2 (ageing or aging)) or (friendl* W/2 (age or ages or aged or aging or ageing or elder*))) AND (intervention* or effect* or evaluat* or impact* or controlled or "control group*" or (before W/5 after) or (pre W/5 post) or "pre-intervention*" or preintervention* or "post-intervention*" or postintervention* or "pre-post” or ((pretest* or "pre test*") and (posttest* or "post test*")) or quasiexperiment* or "quasi experiment*" or "pseudo experiment*" or pseudoexperiment* or "time series” or "time point*" or "repeated measur*") AND ((“active* transport*" or "active* travel*" or ((bicycl* or cycl* or driv* or motorcycl* or walk*) W/2 (coach* or course* or educat* or instruct* or lesson* or school* or train*)) or ((bicycl* or cycli* or driv* or motorcycl* or motorist* or passenger* or pedestrian* or rider* or walk* or traffic* or transport* or automobile* or bus* or car or cars or carpark* or "dial a ride” or garage* or metro or railway* or parking* or sidewalk* or subway* or taxi* or train or trains or underground or vehicle* or crossing* or highway* or lane or lanes or road or roads or street*) W/2 (abilit* or access* or afford* or behaviour* or behavior* or capacit* or danger* or difficult* or ergonomic* or habit* or safe* or stable or stability or unsafe or ((fare* or price* or ticket*) W/2 (discount* or free or reduc*)) or accident* or collision* or crash* or hazard* or incident* or speed*)) or ((bicycl* or cycli* or driv* or motorcycl* or automobile* or bus* or car or cars or metro or railway* or subway* or taxi* or train or trains or underground or vehicle*) W/2 (adapt* or alteration* or modif* or support or usability))) OR ((ambient* W/2 assist* W/2 living) or ((smart or automat*) W/2 home*) or (smart W/2 environment*) or ((home* or hous* or residen* or accommodation* or bath* or bedroom* or kitchen* or room* or shower* or stair* or toilet*) W/2 (access* or adapt* or alteration* or modif* or renovat* or support or usability)) or ((elevator* or handrail* or "hand rail*" or lift or lifts or ramp or ramps or gerontechnolog* or "voice assistant*" or (service W/2 robot*) or ((assist* or intelligen* or safe* or "self-help*" or welfare*) W/2 (aid or aids or device* or platform* or robot* or solution* or technolog*))) and (home* or hous* or residen* or accommodation* or city or cities or communit* or environment* or neighb* or rural or societ* or town* or urban or village*))) OR (((urban or rural) W/2 (plan* or develop* or design*)) or ((built or green or neighb* or street* or rural or urban or cycling or driving or walking) W/2 environment*) or ((neighb* or rural or urban) W/2 space*) or amenit* or "communit* environment*" or greener* or greenness or greenspace* or greenway* or ((green or blue) W/2 (area* or space*)) or "land use*" or streetscape* or (street* W/2 connect*) or "rural renewal*" or "urban renewal*" or "rural form*" or "urban form*" or walkabilit* or ((population or residential or retail or rural or urban) W/2 densit*) or ((aesthetic* or garden or gardens or park or parks or ((plan* or develop* or design*) W/2 (facility or facilities)) or ((outdoor* or public) W/2 (space* or building* or facility or facilities)) or ((indoor* or living or natural or objective or outdoor* or perceived or physical) W/2 environment*)) and (home* or hous* or residen* or accommodation* or city or cities or communit* or neighb* or rural or societ* or town* or urban or village*)) or ((neighb* W/2 (space* or building* or facility or facilities)) and (home* or hous* or residen* or accommodation* or city or cities or communit* or rural or societ* or town* or urban or village*)) or ((communit* W/2 (space* or building* or facility or facilities)) and (home* or hous* or residen* or accommodation* or city or cities or neighb* or rural or societ* or town* or urban or village*)) or (infrastructure* and (city or cities or communit* or cycling or driving or neighb* or pedestrian or public or rural or town* or transport* or urban or village*))))) AND (EXCLUDE (SUBJAREA,"COMP”) OR EXCLUDE (SUBJAREA,"BIOC”) OR EXCLUDE (SUBJAREA,"MATH”))	4335

**Figure:**
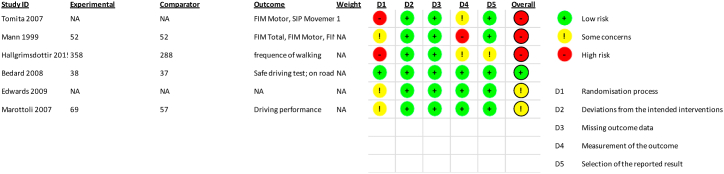


**Figure:**
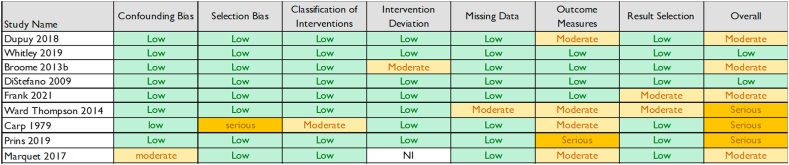


## Appendix 1

### MEDLINE Ovid search strategy

© [2024] World Health Organization; licensee Elsevier.
